# Acute Kahweol Treatment Attenuates Traumatic Brain Injury Neuroinflammation and Functional Deficits

**DOI:** 10.3390/nu11102301

**Published:** 2019-09-27

**Authors:** Hung-Fu Lee, Jhih Syuan Lin, Che-Feng Chang

**Affiliations:** 1 Department of Neurosurgery, Cheng Hsin General Hospital, Taipei 11220, Taiwan; ufae0073@ms7.hinet.net (H.-F.L.); xup5@cycu.org.tw (J.S.L.); 2 Graduate Institute of Physiology, College of Medicine, National Taiwan University, Taipei 10051, Taiwan

**Keywords:** coffee, coffee diterpene kahweol, traumatic brain injury, neuroinflammation, leukocyte infiltration, innate immunity

## Abstract

Traumatic brain injury (TBI) affects millions worldwide with devastating long-term effects on health and cognition. Emerging data suggest that targeting the immune response may offer promising strategies to alleviate TBI outcomes; kahweol, an anti-inflammatory diterpene that remains in unfiltered coffee, has been shown to be beneficial in neuronal recovery. Here, we examined whether kahweol could alleviate brain trauma-induced injury in a mouse model of TBI and its underlying mechanisms. TBI was induced by controlled cortical impact (CCI) and various doses of kahweol were intraperitoneally administered following injury. Contusion volume, brain edema, neurobehavioral deficits, and protein expression and activity were evaluated in both short-term and long-term recovery. We found that kahweol treatments significantly reduced secondary brain injury and improved neurobehavioral outcomes in TBI mice. These changes were accompanied by the attenuation of proinflammatory cytokine secretion, decreased microglia/macrophage activation, and reduction of neutrophil and leukocyte infiltration. In addition, continuous kahweol treatment further improved short-term TBI outcomes compared to single-dosage. Collectively, our data showed that kahweol protects against TBI by reducing immune responses and may serve as a potential therapeutic intervention for TBI patients.

## 1. Introduction

Traumatic brain injury (TBI) is one of the leading causes of death and life-long disability in young adults. Millions of people worldwide seek medical care for this devastating condition that results when accidental external forces apply mechanical energy to the head [1]. TBI initiates from the tearing, stretching, and shearing that cause irreversible damage to the brain parenchyma and subsequent CNS cell death [2]. Following the primary insult, damage-associated molecular patterns (DAMPs) are released from the dying cells causing a cascade of metabolic, biochemical, and inflammatory changes that included the activation of the microglia and peripheral leukocytes. The elicited immune responses further triggered acute neuroinflammation and brain edema, leading to secondary brain injury [3,4,5,6].

The secondary pathological events can last days, weeks, and even months after TBI, which contributes to the long-term neurological deficits oftentimes observed in patients [7]. Hence, strategies that prevent the development of secondary neuroinflammation by rapidly reducing detrimental immune responses are particularly useful for promoting recovery from TBI [8,9]. Although many therapeutic agents have been tested in preclinical models for treating TBI, only a handful show potential to diminish brain damage [9,10,11,12,13,14] and none has yet shown significant improvement in long-term neurobehavioral outcomes in clinical trials [15]. Consequently, a search for other pharmacological targets to promote and accelerate brain recovery after TBI is of dire need.

Coffee, being one of the most widely consumed pharmacologically active beverages, has been shown to hold beneficial effects on reducing the risk of stroke, cardiovascular diseases, and age-related neurodegenerative disorders [16,17,18]. The most abundant bioactive compounds in coffee include caffeine, diterpenes, and polyphenols. Among them is kahweol, an anti-inflammatory diterpene that remains in unfiltered coffee beverages including espresso, Scandinavian boiled, and Turkish coffees [19]. Aside from the well-established anti-carcinogenic property [20,21,22,23,24,25,26], studies on human neuroblastoma and dopaminergic neurons have suggested a neuroprotective function of kahweol [27,28]. In addition, kahweol has been shown to reduce LPS-induced macrophages proinflammatory activation [29] and prevent hepatic injury through its anti-inflammatory property [30,31]. We and others have previously demonstrated that modulation of microglia activation and leukocyte infiltration protect against TBI-induced neuroinflammation and brain injury [32]. To this end, we set out to determine whether the anti-inflammatory property of kahweol helps ameliorate brain trauma-induced injury. We hypothesized that kahweol treatment after TBI would attenuate CNS immune responses and subsequently reduce neuronal injury and promote improved neurobehavioral outcomes.

## 2. Materials and Methods

### 2.1. Animals

All procedures were conducted under the approval of the Institutional Animal Care and Use Committee at Cheng Hsin General Hospital (animal permit number CHIACUC-103-07 and CHIACUC-104-15), and all animals were cared for in accordance with the Guide for the Care and Use of Laboratory Animals published by the U.S. National Institutes of Health (NIH Publication No. 85–23, revised 1996) and ARRIVE guidelines (Animal Research: Reporting In Vivo Experiments). Eight- to ten-week-old male C57BL/6 wild-type (WT) mice were purchased from BioLASCO (Taipei, Taiwan). The animals were bred in groups at 22–26 °C, 35–60% humidity in a 12-h light/dark cycle controlled room with ad libitum access to pellet chow diet and water. A total of 218 mice were used in this study.

### 2.2. Experimental Design

The mice were randomly assigned to different experimental groups. Forty-three TBI mice treated with vehicle or various kahweol dose regimens were used for brain water content measurement; another fifteen mice were used for cytokine array and ELISA analysis after TBI; a total of 160 TBI mice treated with vehicle or various kahweol dose regimens were used for histological analysis and neurobehavioral tests. No mice were excluded from the final statistical analysis.

### 2.3. TBI

A controlled cortical impact (CCI) procedure was carried out, with minor modifications, to induce TBI in mice [32,33]. Briefly, mice were anesthetized with sodium pentobarbital via intraperitoneal injection (65 mg/kg; SCI Pharmtech, Taiwan). The mice were then positioned on a stereotaxic frame, and a midline scalp incision was made to expose the skull. We performed a 5 mm craniotomy on the right parietal cortex, centered on the bregma and 3 mm lateral to the midline. The TBI was induced by impacting the brain surface at the center of craniotomy with a 2.5 mm diameter rounded metal tip at a velocity of 4 m/s and a deformation depth of 2 mm by using a CCI device (eCCI Model 6.3; Custom Design, USA). In the sham animals, the impact step was replaced by a light touch of the dura with the rounded metal tip. After surgery, the mice were placed in a heated cage to maintain body temperature until full recovery from anesthesia.

### 2.4. Kahweol Treatment

Kahweol (Cayman chemical, MI, USA), dissolved in dimethyl sulfoxide (DMSO; Sigma-Aldrich, MO, USA), or a corresponding volume of vehicle (100% DMSO), was intraperitoneally administered immediately after TBI induction. All solutions were freshly and aseptically prepared. In the single dosage injection experiment, we randomly distributed TBI mice to receive either vehicle or different doses (5, 10, 20 mg/kg) of kahweol. In the multiple-dosage experiments, the mice were given the first injection of 5 mg/kg kahweol immediately after TBI and then treated with 1 mg/kg kahweol every 24 h.

### 2.5. Tissue Processing and Histology

Mice were euthanized at various time points after TBI by transcardial perfusion with PBS followed by 4% paraformaldehyde. All solutions were maintained at pH 7.4 and 4 °C. Brains were collected and post-fixed in 4% paraformaldehyde overnight and then transferred to 30% sucrose in PBS for cryoprotection. Coronal cryosections (20 µm thick) from the level of the olfactory bulbs to the visual cortex were used for cresyl violet histology. Another set of coronal sections (10 µm thick) around the traumatic level was used for Fluoro-Jade C (FJC) staining and immunolabeling.

### 2.6. Contusion Volume Assessment

Contusion volume was quantified using cresyl violet-stained sections at 20 rostral-caudal levels that were spaced 400 μm apart. We digitized the sections and analyzed them using a 1.25x objective and ImageJ software (NIH, Bethesda, MD, USA). The contusion volume in cubic millimeters was computed by summation of the damaged areas multiplied by the interslice distance (400 µm) as previously described [32].

### 2.7. Measurements of Degenerating Neurons, Macrophage/Microglia Activation, and Neutrophil and Leukocyte Infiltration

We performed FJC staining as well as Iba-1, Ly6G, and CD45 immunolabeling to detect degenerating neurons, microglia/macrophage activation, and neutrophil and leukocyte infiltration in the TBI brains, respectively [34]. For FJC staining, cryosections were rehydrated in solutions of 1% NaOH in 80% ethanol, 70% ethanol, and distilled water for 5 min each. Slides were then incubated in 0.06% KMnO4 for 15 min, washed in distilled water for 1 min, and incubated in a 0.0001% solution of FJC (Merck Millipore, Burlington, MA, USA) for 10 min. Sections were observed and imaged under a fluorescence microscope (Olympus BX-51, Japan) with blue excitation light (450–490 nm). Immunofluorescent assessment was performed by incubating the sections with anti-mouse Iba-1, (1:500; 019-19741, Wako, Japan) or anti-mouse Ly6G (1:500; ab25377, Abcam, UK) primary antibodies overnight at 4 °C, followed by the appropriate secondary antibodies, goat anti-rabbit conjugated with rhodamine (TRITC) at 1:500 (111-025-003, Jackson Immuno Research Inc. Bellefonte, PA, USA) or goat anti-rat at 1:500 conjugated with Fluorescein (FITC) (115-095-003, Jackson Immuno Research Inc., Bellefonte, PA, USA) for 2 h at room temperature. All sections were observed and imaged by a blinded researcher under a fluorescence microscope (Olympus BX-51). For CD45 immunohistochemical staining, sections were rehydrated and quenched with 0.3% H_2_O_2_ for 10 min. After blocking with 10% normal goat serum (Jackson Immuno Research Inc., Bellefonte, PA, USA), sections were incubated with anti-mouse CD45 primary antibody (550539, BD pharmingen, San Diego, CA, USA) overnight at 4 °C, followed by goat anti-rat (1:1000; 112-065-003, Jackson Immuno Research Inc., Bellefonte, PA, USA) secondary antibody incubation for 2 h at room temperature. The signal of the secondary antibody was amplified with VECTASTAIN Elite ABC Kit (Vector Laboratories, Carlsbad, CA, USA). The color of the sections was developed with the DAB Enhanced Liquid Substrate System (Sigma-Aldrich, Saint Louis, MO, USA) and counterstained with creysl violet. After dehydration, sections were mounted, observed, and imaged by a blinded researcher.

### 2.8. Quantification of FJC, Iba-1, Ly6G, and CD45 Staining

FJC, Iba-1, Ly6G, and CD45 staining was quantified on sections at 0.74 mm anterior to the bregma. Three sections per animal were imaged using a fluorescence microscope by a blinded observer. Positive cells were counted by sampling an area (820 × 610 μm^2^) immediately adjacent to the cortical contusion margin in four randomly selected, non-overlapping fields using a magnification of 200x. Iba-1-positive activated microglia/macrophages were defined based on a combination of morphological criteria (short, thick processes) and a cell body diameter larger than 7.5 μm [34]. The total number of FJC, Iba-1, Ly6G, and CD45-positive cells was expressed as cells/mm^2^.

### 2.9. Cytokine Array and ELISA

Mice were anesthetized and decapitated at day 1 after CCI or sham procedure and a 3-mm coronal section from the injured area over the right parietal cortex was collected. The collected tissues were rapidly frozen in liquid nitrogen, and stored at −80 °C until use. We homogenized tissues in ice-cold protein extraction reagent (T-PER Reagent; Pierce, Billerica, MA, USA) with protease inhibitor cocktails (Sigma-Aldrich, MO, USA). Total protein was quantified by the Bradford protein assay (Bio-Rad, Richmond, CA, USA). Parallelly for the determination of the relative levels of selected mouse cytokines and chemokines in the vehicle- and kahweol-treated TBI brains, the tissue supernatants were screened using Proteome Profiler Mouse Cytokine Array Panel A (ARY006, R&D Systems, Inc., Minneapolis, MN, USA) according to the manufacturer’s instructions. A total of 900 μL tissue supernatant was used for each nitrocellulose membrane. Each replicate includes supernatants from three individual animals. The intensity of the immunoreaction developed was assessed densitometrically using ImageJ software (NIH, Bethesda, MD, USA). In order to confirm the cytokines/chemokines levels from the array screening, IL-1β, MIP-1α, MIP-2, and TMIP-1 were quantified from equal amounts of protein by ELISA (R&D Systems, Inc., Minneapolis, MN, USA) according to the manufacturer’s instructions. All samples and standards were assayed in duplicates. Tissue cytokines/chemokines concentrations were expressed as pg/mg protein.

### 2.10. Brain Water Content Measurement

Brain water content was measured in a 3 mm coronal tissue section of the ipsilateral hemisphere that contained TBI territory. Brain samples were weighed to obtain the wet weight prior to dry at 100 °C for 24 h. The dry weight was measured after this dehydration procedure. Brain edema was evaluated by measuring brain water content using the formula of [(wet weight − dry weight)/wet weight] × 100 [35,36].

### 2.11. Neurologic Function Evaluation

A researcher blinded to the treatment assignments evaluated functional and neurological deficits of TBI mice by modified neurological severity score (mNSS), beam score test, beam walk test, vibrissae-elicited forelimb placing test, and rotarod test as previously described [32,33,34,37,38]. First, the sensory, motor, balance, and reflex of mice were evaluated by the modified neurological severity score (mNSS) as described in detail previously in [33]. Neurologic deficit was graded on a scale of 0–18 from the normal to maximal deficit. The beam score test reflects the active balance by hindlimb performance as mice traversed along a narrow, elevated beam (122.0 cm × 2.5 cm). Mice were scored based on a 1 to 7 rating scale where a lower score reflected greater severity of injury [39]. The beam walk test was used to assess fine motor coordination and function by measuring the ability of mice to traverse an elevated beam. The time that mice took to cross the beam was recorded and the maximal observation time was 60 s for mice that failed to cross the beam. The longer time represents a more severe injury. For the vibrissae-elicited forelimb placing test, the mice were allowed to use all four limbs to hang freely by the edge of table, meanwhile the vibrissae of mice were brushed. Intact mice placed their ipsilateral forelimb to the table top quickly. A total of 10 trials were recorded each day and the data were calculated as a percentage of successful placing responses out of all trials. The rotarod test was used to measure motor function and balance. Mice were placed on an accelerating rotarod cylinder with slowly increasing speed, from 6 to 42 rpm over 7 min. The trial was terminated once the mice were no longer able to keep walking on the rungs. The data were recorded as the amount of time that mice remained on the rotarod.

### 2.12. Statistical Analysis

All data are presented as mean ± standard deviation (SD). We evaluated all neurobehavioral outcomes by two-way repeated measure analysis of variance (ANOVA) to detect significant differences between and among treatment groups. For histological, anatomical, and biochemical tests, we performed one-way or two-way ANOVA for comparisons among multiple groups. Bonferroni post-hoc analysis was used to determine where those differences occurred. Differences between two groups were tested with the Student’s t-test (Systat Software Inc.). A *p* value less than 0.05 was considered as statistically significant.

## 3. Results

### 3.1. Kahweol Reduces TBI-Induced Early Brain Injury, Brain Edema, and Neurologic Deficits

**Figure 1:**
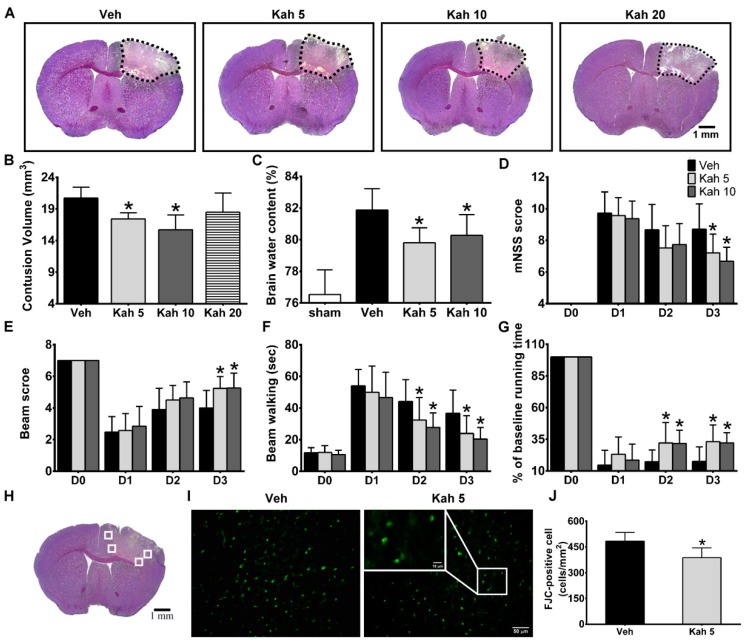
Kahweol treatment improved acute TBI outcomes. (**A**) Representative cresyl violet-stained brain sections of kahweol-treated TBI animals. Dotted area indicates contusion region that lacks staining in the ipsilateral cortex at day 3 post-injury. Scale bar = 1 mm. (**B**) When compared with Veh group, Kah 5- and Kah 10-treated groups significantly had smaller contusion volume. There was no significant change in brain contusion volume between Veh- and Kah 20-treated groups. *n* = 10 for Veh, Kah 5, and Kah 10 groups and *n* = 6 for Kah 20 group. *p < 0.05 versus vehicle group. (**C**) Brain water content was reduced by Kah 5 and Kah 10 treatment at day 3 post-injury. *n* = 11 for sham group, *n* = 10 for Veh and Kah 5 groups, and *n* = 12 for Kah 10 group. * *p* < 0.05 versus vehicle group. In addition, Kah 5 and Kah 10 significantly reduced (**D**) modified neurological severity score (mNSS) at day 3 post-CCI, improved (**E**) beam score on day 3, reduced (**F**) the beam walk latencies at days 2 and 3, and improved (**G**) the rotarod performance at days 2–3 post-injury. *n* = 21 for Veh and Kah 5 groups and *n* = 19 for Kah 10 group. * *p* < 0.05 versus vehicle group. (**H**) Representative cresyl violet-stained TBI brain section shows areas (white box) selected for Fluoro-Jade C (FJC)-positive cell counting. Scale bar = 1 mm. (**I**) Representative FJC-stained sections of a vehicle-treated and Kah 5-treated animal at three days post-injury. Inset shows FJC-positive cells at a higher magnification. Scale bar = 50 μm in the full image and 10 μm in the inset. (**J**) Quantification shows that Kah 5 significantly reduced the number of degenerating neurons at three days post-injury. *n* = 5 for Veh group and *n* = 6 for Kah 5 and Kah 10 groups. * *p* < 0.05 versus the Veh group. Throughout, data are the mean ± SD. Veh, vehicle; Kah 5, kahweol 5 mg/kg; Kah 10, kahweol 10 mg/kg; Kah 20, kahweol 20 mg/kg.

To determine the optimal effective dose of kahweol (Kah) treatment in TBI, we first administered different doses of Kah to mice subjected to the CCI procedure. Contusion volume and brain edema were assessed at day 3 after TBI as CCI-induced brain damage usually peaks around this time [32,35,36]. Brain sections stained with cresyl violet revealed cortical cell loss and brain lesions were identified by a lack of color (Figure 1A). The contusion volume was smaller in mice that received 5 mg/kg Kah (Kah 5; 17.63 ± 1.08 mm^3^; *p* = 0.03) or 10 mg/kg Kah (Kah 10; 15.71 ± 2.34 mm^3^; *p* < 0.001) than in those that received vehicle treatment (Veh; 20.70 ± 1.75 mm^3^). Contusion volume did not differ between mice that received vehicle and those that received 20 mg/kg Kah (Kah 20; 18.47 ± 3.02 mm^3^; *p* = 0.339) or between Kah 5 and Kah 10 groups (*p* = 0.322; Figure 1B). No obvious brain contusion was observed in the sham-operated group (data not shown). As brain edema has a critical impact on TBI prognosis [40], we further measured the brain water content to evaluate edema. Brain water content was significantly decreased in the ipsilateral hemisphere in mice treated with either Kah 5 (79.8 ± 0.95%; *P* = 0.007) or Kah 10 (80.3 ± 1.31%; *p* = 0.044) when compared with the vehicle-treated group (81.9 ± 1.35%) (Figure 1C).

A battery of neurobehavioral tests was performed at days 1, 2, and 3 after TBI to determine the effect of Kah on acute neurologic deficits. When compared with vehicle (Veh), Kah 5 and Kah 10 both showed a significant improvement on neurological severity score (mNSS) (Veh 8.71 ± 1.59 vs. Kah 5 7.21 ± 1.18; *p* = 0.001; Kah 10 6.68 ± 0.89; *p* < 0.001 on day 3) (Figure 1D), beam score (Veh 4.00 ± 1.11 vs. Kah 5 5.24 ± 0.75; *p* = 0.005; Kah 10 5.26 ± 0.93; *p* = 0.002 on day 3) (Figure 1E), beam walking (Veh 44.14 ± 13.84 s vs. Kah 5 32.36 ± 14.26 s; *p* = 0.046; Kah 10 27.71 ± 9.22 s; *p* < 0.001 on day 2; Veh 36.69 ± 14.53 s vs. Kah 5 23.89 ± 11.28 s; *p* = 0.041; Kah 10 20.38 ± 7.24 s; *p* < 0.001 on day 3) (Figure 1F), and rotarod test (Veh 17.2 ± 9.21% vs. Kah 5 32.0 ± 16.3%; *p* < 0.001; Kah 10 31.6 ± 10.6%; *p* < 0.001 on day 2; Veh 17.6 ± 11.4% vs. Kah 5 33.1 ± 13.1%; *p* < 0.001; Kah 10 32.2 ± 7.9%; *p* < 0.001 on day 3) (Figure 1G) from day 2 or 3 after TBI.

To determine whether the reduction in early brain injury and the improvement of neurologic deficits in Kah-treated TBI mice correlated with reduced neuronal degeneration, we performed FJC staining on brain sections after Veh or Kah treatment (Figure 1H,I). In addition, because Kah 5 significantly reduced contusion volume, brain edema, and neurologic deficits, we used Kah 5 to further explore the therapeutic efficacy of Kah treatment and performed mechanistic studies in later experiments. FJC were evident at day 3 after TBI adjacent to the lesion area. Compared to Veh treatment, Kah 5 reduced the number of FJC-positive cells (from 483.55 ± 51.05 cells/mm^2^ to 388.33 ± 55.93 cells/mm^2^; *p* = 0.014) (Figure 1J), suggesting Kah 5 treatment reduced neuronal degeneration.

### 3.2. Kahweol Reverses Long-Term Brain Injury and Neurobehavioral Functions

**Figure 2:**
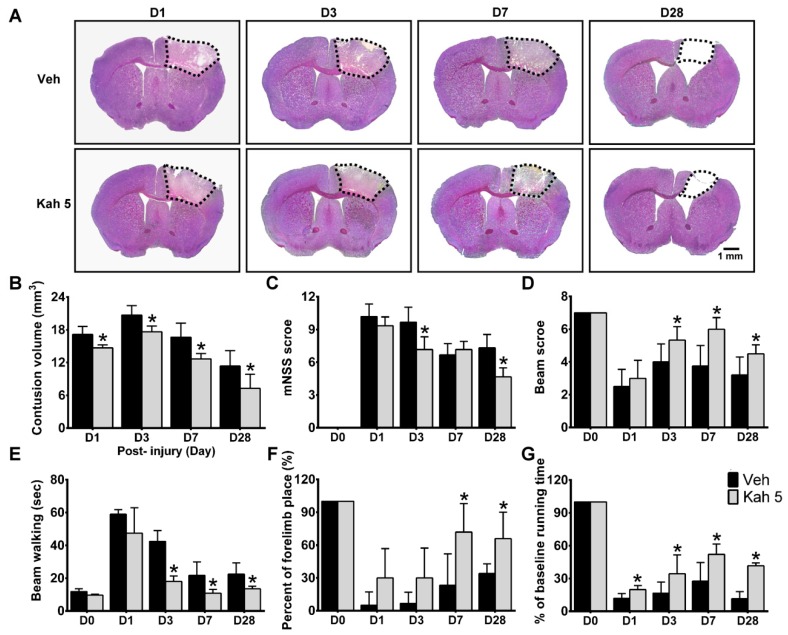
Single-dose kahweol treatment reduced long-term brain tissue loss and improved long-term neurobehavioral functions after TBI. (**A**) Representative cresyl violet-stained brain sections of Veh- and Kah 5-treated TBI animals at days 1, 3, 7, and 28 post-injury. Dotted area indicates the contusion region that lacks staining in the ipsilateral cortex. Scale bar = 1 mm. (**B**) Kah 5 treatment significantly reduced contusion volume from days 1 to 28 after TBI. *n* = 6 to 10 for Veh and Kah 5 groups. *p < 0.05 versus Veh group. When compared with the Veh group, Kah 5-treated animals showed significantly reduced (**C**) modified neurological severity score (mNSS) on days 3 and 28 post-TBI, better (**D**) beam score, and reduced (**E**) beam walk latencies from days 3 to 28 post-injury, improved (**F**) forelimb function from days 7 to 28, and enhanced (**G**) rotarod performance. *n* = 4 to 8 per group. * *p* < 0.05 versus Veh group. Throughout, data are the mean ± SD. Veh, vehicle; Kah 5, kahweol 5 mg/kg.

In clinical, TBI can lead to secondary neurologic problems that develop and persist months to years after initial injury [41]. Preclinical guidelines has also appealed that the assessment of long-term outcomes is crucial for assessing pharmacological therapies for TBI [15]. We therefore examined the contusion volume and neurobehavioral functions in the Veh- and Kah 5-treated mice over the course of 28 days after TBI at selected timepoints (Figure 2A). When compared with the Veh group, Kah 5 mice had decreased brain tissue damage (Veh 17.15 ± 1.46 mm^3^ vs. Kah 5 14.69 ± 0.57 mm^3^; *p* = 0.001 on day 1; Veh 20.70 ± 1.75 mm^3^ vs. Kah 5 17.63 ± 1.08 mm^3^; *p* < 0.001 on day 3; Veh 16.65 ± 2.59 mm^3^ vs. Kah 5 12.66 ± 1.00 mm^3^; *p* = 0.028 on day 7; Veh 11.36 ± 2.81 mm^3^ vs. Kah 5 7.27 ± 2.59 mm^3^; *p* = 0.004 on day 28) (Figure 2B). Kah 5 mice also showed enhanced performance in the mNSS (Veh 9.67 ± 1.37 vs. Kah 5 7.17 ± 1.17; *p* = 0.002; on day 3; Veh 7.33 ± 1.21 vs. Kah 5 4.67 ± 0.82; *p* < 0.001; on day 28), beam score (Veh 4.00 ± 1.10 vs. Kah 5 5.33 ± 0.82; *p* = 0.038; on day 3; Veh 3.75 ± 1.26 vs. Kah 5 6.00 ± 0.71; *p* = 0.014; on day 7; Veh 3.20 ± 1.10 vs. Kah 5 4.50 ± 0.55; *p* = 0.03; on day 28), beam walking (Veh 42.49 ± 6.65 s vs. Kah 5 17.99 ± 3.43 s; *p* < 0.001; on day 3; Veh 21.68 s ± 8.21 s vs. Kah 5 10.80 s ± 2.43 s; *P* = 0.035; on day 7; Veh 22.45 s ± 6.95 s vs. Kah 5 13.50 s ± 1.56 s; *p* = 0.012; on day 28), forelimb placing (Veh 23.33 ± 28.75% vs. Kah 5 72.0 ± 25.88%; *p* = 0.017; on day 7; Veh 34.0 ± 8.94% vs. Kah 5 66.0 ± 24.08%; *p* = 0.024; on day 28) and rotarod test (Veh 11.8 ± 4.42% vs. Kah 5 19.9 ± 3.68%; *p* = 0.001; on day 1;Veh 16.6 ± 10.1% vs. Kah 5 34.4 ± 17.1%; *p* = 0.017; on day 3; Veh 27.6 ± 17.1% vs. Kah 5 52.1 ± 9.4%; *p* = 0.001; on day 7; Veh 11.6 ± 6.4% vs. Kah 5 41.7 ± 2.5%; *p* < 0.001; on day 28) (Figure 2C–G) until at least 28 days after TBI. Taken together, these findings show that Kah 5 can reduce long-term brain damage and neurobehavioral deficits following TBI.

### 3.3. Kahweol Exerts An Acute Anti-Inflammatory Effect in the TBI Brain

**Figure 3:**
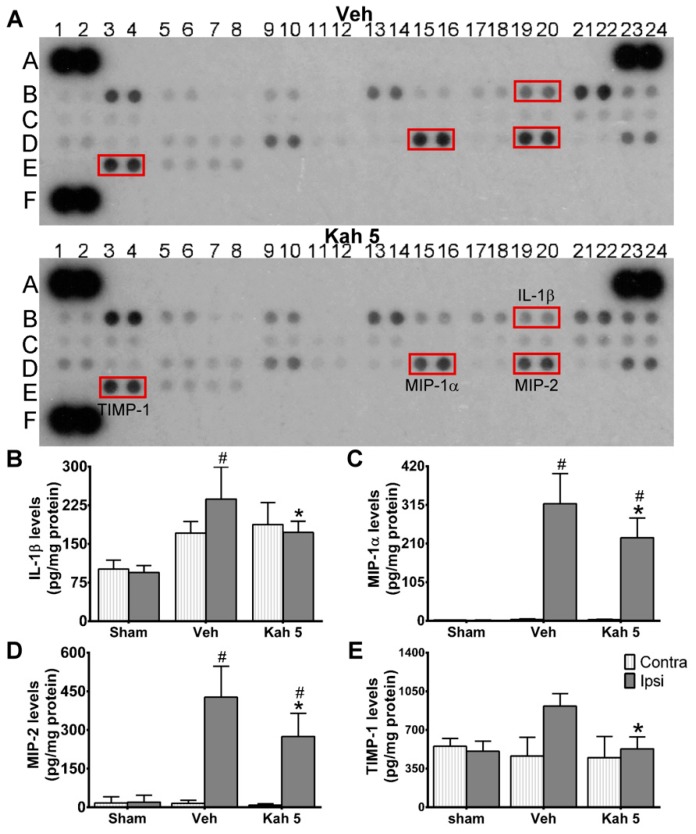
Kahweol treatment attenuated proinflammatory cytokines/chemokines secretion levels after TBI. (**A**) Representative chemiluminescence images of proteome profiler array from Veh- and Kah 5-treated TBI animals at 1-day post-injury. The red box indicates selected candidate proteins that were reduced by Kah 5 treatment. A1.2, A23.24, and F1.2 were the loading controls in the panels. *n* = 2 per group; each independent experiment includes three individual animals. Quantification analysis showed that increased levels of (**B**) IL-1β, (**C**) MIP-1α, (**D**) MIP-2, and (**E**) TIMP-1 in the vehicle group were markedly reduced in the Kah 5-treated day 1 TBI animals. *n* = 3 for sham group and *n* = 6 for Veh and Kah 5 groups. * *p* < 0.05 versus Veh group. ^#^ *p* < 0.05 versus Contra group. Data are the mean ± SD. Veh, vehicle; Kah 5, kahweol 5 mg/kg; Contra, contralateral; Ipsi, ipsilateral.

To begin to elucidate the possible mechanism by which Kah provides neuroprotection, we screened 40 mouse cytokines, chemokines, and acute phase proteins in mouse brains one day after TBI using a cytokine array. We observed that interleukin-1 beta (IL-1β, B19,20), macrophage inflammatory protein 1-alpha (MIP-1α, D15,16), macrophage inflammatory protein 2 (MIP-2, D19,20), and tissue inhibitors of metalloproteinases 1 (TIMP-1, E3,4) in TBI brain tissue extracts were diminished after Kah treatment (Figure 3A). The initial screening result was further confirmed by enzyme-linked immunosorbent assay (ELISA) in which IL-1β (237.02 ± 62.21 pg/mg protein vs. 172.14 ± 21.63 pg/mg protein; *p* = 0.037), MIP-1α (318.37 ± 81.70 pg/mg protein vs. 226.19 ± 53.12 pg/mg protein; *p* = 0.019), MIP-2 (427.03 ± 120.06 pg/mg protein vs. 274.99 ± 90.29 pg/mg protein; *p* = 0.008), and TIMP-1 (916.39 ± 252.68 pg/mg protein vs. 529.70 ± 242.80 pg/mg protein; *p* = 0.039) protein levels were significantly decreased after Kah treatment at day 1 post-TBI (Figure 3B–E).

### 3.4. Kahweol Treatment Dampens Immune Cells Activation and Invasion

**Figure 4:**
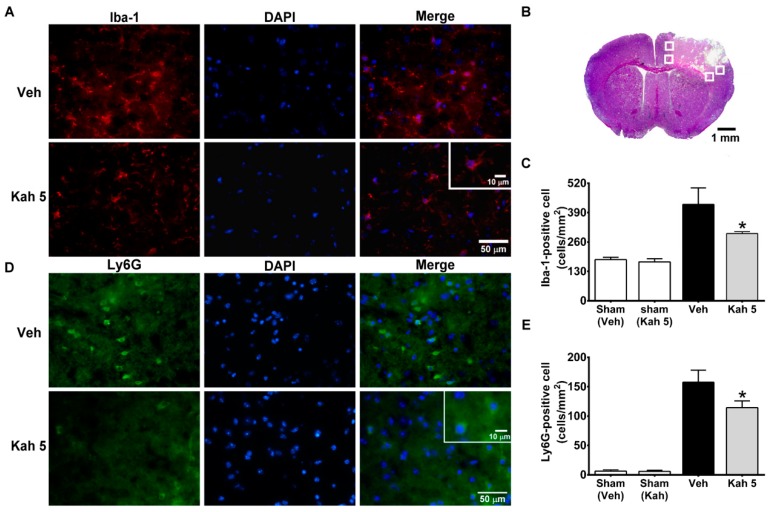
Kahweol treatment reduced neuroinflammation after TBI. (**A**) Representative immunofluorescence of microglia/macrophage marker Iba1 (red) and nuclear marker DAPI (blue) in the Veh- and Kah 5-treated animals at three days following TBI. The inset represents higher magnification of the Iba1-positive cells. Scale bar = 50 μm in the full image and 10 μm in the inset. (**B**) Representative cresyl violet-stained TBI brain section showed areas (white box) selected for double-positive cell counting. Scale bar = 1 mm. (**C**) Quantification of Iba1-positive cells showed that increased levels of inflammatory microglia/macrophage in the Veh-treated group (compared versus sham-injured group) were attenuated in Kah 5-treated animals 3 days post-injury. *n* = 3 per group. * *p* < 0.05 versus Veh group. (**D**) Representative immunostaining of neutrophils marker Ly6G (green) and nuclear marker DAPI (blue) in the Veh- and Kah 5-treated animals at three days following TBI. The inset image shows the Ly6G-positive cells at a higher magnification. Scale bar = 50 μm in the full image and 10 μm in the inset. (**E**) Increased levels of neutrophils infiltration in the vehicle-treated group were reduced in the Kah 5-treated group. *n* = 3 mice for the sham-groups, *n* = 4 for the Veh group, and *n*= 5 for the Kah 5 group. * *p* < 0.05 versus the Veh group. Throughout, data are mean ± SD. Veh, vehicle; Kah 5, kahweol 5 mg/kg; Iba-1, ionized calcium-binding adapter molecule 1; Ly6G, Lymphocyte antigen 6 complex locus G6D.

**Figure 5:**
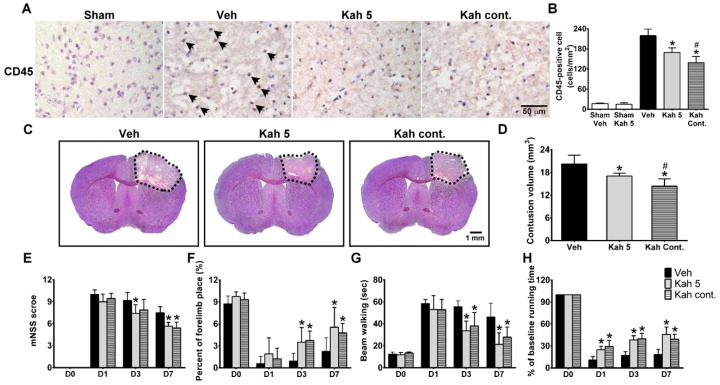
Multiple-dose kahweol treatment reduced brain injury in acute phase following TBI, but did not affect long-term neurobehavioral functions. (**A**) Representative IHC staining of leukocyte marker CD45 in the sham-injured, Veh-treated, and Kah 5- and Kah cont.-treated TBI animals. Scale bar = 50 μm. (**B**) Quantification of CD45-positive cells showed that increased levels of leukocyte infiltration in the Veh-treated group (compared versus sham-injured group) were markedly reduced in Kah 5-treated animals at three days post-injury. Multiple-dose Kah treatment further attenuated leukocyte infiltration compared to single-dose treatment in acute phase following injury. *n* = 3 per group. * *p* < 0.05 versus the Veh group. ^#^ *p* < 0.05 versus Kah 5 group. (**C**) Representative cresyl violet-stained brain sections of Veh-, Kah 5-, and Kah cont.-treated animals at day 3 following TBI. Dotted area indicates the contusion region in the ipsilateral cortex. Scale bar = 1 mm. (**D**) Continuous treatment of Kah further reduced contusion volume in the Kah cont. group compared to the Kah 5 group at day 3 post-injury. *n* = 6 for Veh group and *n* = 5 for Kah 5 and Kah cont. groups. * *p* < 0.05 versus the Veh group. ^#^ *p* < 0.05 versus Kah 5 group. Kah cont. treatment reduced (**E**) modified neurological severity score (mNSS) at day 7 post-injury, improved the (**F**) forelimb functions at days 3 and 7, reduced the (**G**) beam walk latencies at days 3 and 7, and improved the (**H**) rotarod performance at days 1, 3, and 7 compared to vehicle post-injury. However, there were no significant differences in neurobehavioral outcomes between the Kah 5- and Kah cont.-treated groups. *n* = 6 to 12 per group. * *p* < 0.05 versus the Veh group. Throughout, the data are mean ± SD. Veh, vehicle; Kah 5, kahweol 5 mg/kg; Kah cont., multiple-dose kahweol.

Our screening suggested that the cytokines/chemokines released during the acute phases following TBI were modulated by kahweol treatment and are highly relevant to immune cell activation and infiltration in the TBI brain [42,43]. We next sought to assess in depth the effect of kahweol treatment on microglia/macrophage activation, and neutrophil and leukocyte infiltration. Iba-1-positive activated microglia/macrophages (Figure 4A) were observed within the injured cortex (Figure 4B) at day 3 after TBI, and the number of Iba-1-positive cells was significantly reduced in Kah 5 mice (297.33 ± 8.82 cells/mm^2^; *p* = 0.019) compared with Veh mice (426.00 ± 73.52 cells/mm^2^) (Figure 4C). Invading neutrophils and leukocytes were evident by Ly6G and CD45 immunofluorescence, respectively (Figure 4D and Figure 5A). As expected, Kah 5 reduced the number of Ly6G- (157.43 ± 20.55 cells/mm^2^ vs. 114.34 ± 11.53 cells/mm^2^; *p* = 0.002) (Figure 4E) and CD45- (231.11 ± 7.13 cells/mm^2^ vs. 175.11 ± 9.67 cells/mm^2^; *p* = 0.001) (Figure 5B) positive cells in the TBI territory compared with the Veh-treated group. Overall, our biochemical and histological analyses suggest that kahweol ameliorates acute immune burden in the TBI brain.

### 3.5. Continuous Kahweol Treatment Reverses Acute But Not Long-Term TBI Outcomes

To further investigate whether continuous treatment of kahweol could advance TBI recovery to an even greater extent, the mice were continuously administered kahweol (hereafter referred as Kah cont.) treatment at a low dose (1 mg/kg) or vehicle every 24 h after the first Kah 5 injection until day 3 or 7 after TBI. We found that continuously treated TBI mice with kahweol showed further reduced leukocyte infiltration (Kah cont.; 138.67 ± 19.01 cells/mm^2^ vs. Kah 5 175.11 ± 9.67 cells/mm^2^; *p* = 0.014) (Figure 5A,B) and brain damage (Kah cont.; 14.38 ± 1.91 cm^3^ vs. Kah 5 17.06 ± 0.77 cm^3^; *p* = 0.022) (Figure 5C,D), when compared with the single-dosage Kah 5-treated group at day 3 after TBI. Interestingly, Kah cont. treatment for seven days did not further the beneficial effect of Kah 5 treatment on the functional performance of TBI mice as evident in mNSS, forelimb placing, beam walking, and rotarod tests (Figure 5E–H).

## 4. Discussion

This work shows several first-time findings: (1) acute single-dosage kahweol treatment ameliorates TBI-induced brain parenchymal damage and reverses short-term and long-term functional outcomes; (2) in adult mice subjected to the CCI model, kahweol treatment reduces neuronal degeneration, neuroinflammation, and leukocyte infiltration; and (3) continuous administration of kahweol treatment advances the protective effects in the acute phase following TBI.

TBI is a leading cause of neurological disability worldwide [44,45]. Currently, no therapies exist that effectively cure the acute motor deficits nor improve long-term neurologic outcomes in TBI patients [15,46,47]. Neuroinflammatory process, a key determinant of TBI outcomes, progresses within minutes after the initial insult and worsens over time [9,41]. Our findings are in agreement with previous studies showing that kahweol reduces the production of pro-inflammatory cytokines and chemokines [20,30]. We further demonstrated that an acute single dose of kahweol treatment confers anti-inflammatory function through the reduction of IL-1β, MIP-1α, MIP-2, and TIMP-1 levels in the TBI brain at day 1 after CCI, and subsequent dampening of microglia/macrophage activation and leukocyte invasion at day 3. This beneficial property of kahweol in turn leads to a decrease in contusion volume and neuronal degeneration, which improved long-term brain injury and neurobehavioral performance in TBI mice.

As inflammatory mediators, IL-1β plays a crucial role in regulating CNS inflammation by triggering detrimental microglia/macrophage activation in the TBI brain [48,49]. Mice treated with the IL-1 receptor antagonist show mitigated neuroinflammation and brain volumetric loss after trauma [50]. In addition, higher TIMP-1 level is associated with mortality in TBI patients [51]. The chemoattractants MIP-1α and MIP-2 have been shown to promote neutrophil and leukocyte infiltration in the inflamed tissue [52,53] and therefore correlate to the extent of brain damage after injury [54]. In parallel, our histological observation agrees with the biochemical measurements from the TBI brains. The number of activated microglia/macrophages and invaded neutrophils and leukocytes was reduced in the TBI brain after kahweol treatment. Furthermore, neuroinflammation oftentimes results in brain edema, which is one of the major prognostic factors in treating patients with TBI [2,55,56]. Our study has shown that kahweol treatment reduces brain edema by suppressing harmful neuroinflammation. Together these findings suggest that kahweol treatment facilitates inflammation reduction after TBI.

While a single acute dose of kahweol treatment is protective in TBI mice, interestingly, continuous kahweol treatment reduces contusion volume and leukocyte infiltration in acute phase following TBI, but fails to further improve long-term functional performance at day 7. One possible explanation of this phenomenon is that immune cells, in particular macrophages and T cells, while triggering detrimental neuroinflammation in the acute phase following brain injury, are actually essential for CNS repair at the chronic stage [57,58]. It is likely that the reduction of immune cells infiltration after continuous kahweol treatment may impede the otherwise beneficial brain repair process during the chronic stage after TBI. Additional insights can be gained from distinguishing the different populations amongst leukocytes and identifying microglia/macrophage phenotypic changes after kahweol treatment. Future studies using flow cytometry to analyze the composition and phenotype of immune cells would shed light on the mechanism of how kahweol affects immune cell functions and macrophage polarization.

To our knowledge, our work provides the first in vivo evidence that kahweol reverses TBI-induced brain damage and functional deficits through modulating local and systemic innate immune responses. Since we cannot exclude the possibility of kahweol having a direct protective effect on neurons and performing multiple functions such as anti-oxidative property that subsequently aid in TBI recovery [27,28], further studies are needed to determine the mediators and signaling pathways through which kahweol plays an important role in TBI treatment. While sex difference also affects the course of TBI neuroinflammation [59,60] and only male mice were used in this study, future efforts to discover the therapeutic efficacy of kahweol in female mice following TBI are clearly warranted. A better understanding of the pharmacologic property of kahweol would guide moving forward to the clinical trial.

## 5. Conclusions

In conclusion, our results demonstrate that acute kahweol treatment significantly decreased TBI-induced brain injury and function deficits. This reduction was associated with the downregulation of neuroinflammation and peripheral immune cells invasion. Therefore, kahweol might be a potential novel agent for TBI treatment.
